# The complete mitochondrial genome of *Varuna yui* (Decapoda: Brachyura: Varunidae) and its phylogeny

**DOI:** 10.1080/23802359.2018.1443043

**Published:** 2018-02-23

**Authors:** Fan Lin, Zhuofang Xie, Hanafiah Fazhan, Juliana C. Baylon, Xiaolong Yang, Huaqiang Tan, Mengyun Guan, Xi Shi, Mhd Ikhwanuddin, Hongyu Ma

**Affiliations:** a Guangdong Provincial Key Laboratory of Marine Biotechnology, Shantou University, Shantou, China;; b Division of Biological Sciences, College of Arts and Sciences, University of the Philippines Visayas, Miagao, Philippines;; c Institute of Tropical Aquaculture, Universiti Malaysia Terengganu, Kuala Terengganu, Malaysia

**Keywords:** *Varuna yui*, mitochondrial genome, phylogenetic analysis

## Abstract

The complete mitochondrial genome plays an important role in the research on phylogenetic relationship. Here, we reported the first complete mitochondrial genome sequence of *Varuna yui* Hwang & Takeda, 1986 (Varunidae). The complete mtDNA (15,915 bp in length) consisted of 13 protein-coding genes, 22 tRNAs, two rRNA genes, and a control region. The gene arrangement was identical to those observed in the Varunidae species. The phylogenetic analysis suggested that *V. yui* had close relationship with other Varunidae species (*Helicetient sinensis*, *Eriocher sinesis*, etc.). The newly described genome may facilitate further comparative mitogenomic analysis within Varunidae species.

The sundaic paddle crab (*Varuna yui*) was first identified as a new species in Taiwan (Hwang and Takeda 1986). It is mainly distributed in Indo-West Pacific, from continental shelf waters of the Sunda Shelf to southern China and Philippines (Carpenter and Niem 1998). *Varuna yui* is closely related to the river swimming crab *V. litterata*, which also belongs to Varunidae (Hwang and Takeda 1986; Ng et al. 2008). However, as the delimitation of the family is undergoing revision (Schubart et al. 2006), it remains controversial whether it should be placed in Grapsoidea or Ocypodoidea. The complete mitochondrial genome is useful for deeply understanding the phylogenetic relationships among related species (Yang et al. 2017). However, no complete mitogenome data are available for *Varuna* genus currently. Currently, only COI and partial 16S rRNA sequence of *V. litterata* are available in GenBank database. Here, the first complete mitochondrial genome sequence of *V. yui* was determined.

Specimens of *V. yui* were collected from a commercial market in Tuopu Town (23.4071°N, 116.6588°E), Shantou, China and kept in the Marine Biology Institute, Shantou University, China. Universal primers were used to amplify partial of COIII, ND5, ND4, Cytb (Burger et al. 2007), and 12S rRNA (Xin et al. 2017) sequences. Long PCR and a primer-walking sequencing strategy were applied to obtain the complete mitogenome sequence.

The complete mitogenome sequence of *V. yui* was 15,915 bp in length (Genbank accession number: MG756602) and contained 13 protein-coding genes, 22 tRNA genes, two rRNA genes, and a control region. Of the 37 genes, 23 were encoded by the heavy strand, whereas the others were encoded by the light strand. The gene arrangement was identical to those of the compared Varunidae species, such as *Helicetient sinensis* (Xin et al. 2017) and *Cyclograpsus granulosus* (Tan et al. 2016). The overall nucleotide composition was 35.70% for A, 36.48% for T, 10.22% for G, and 17.59% for C, respectively. Eight protein-coding genes were initiated by ATG. Two genes (ND2 and ND3) were started by ATC. ND1 and ND6 were started by ATA. While, ATP6 was started by ATT. Two kinds of termination codon (TAA and TAG) were identified in eleven coding genes, while two incomplete termination codons (T–) were found in the other two genes (COI and Cytb).

To determine the phylogenetic position of *V. yui*, the phylogenetic tree was constructed using the concatenated sequences of 12 coding genes (except ND6) from 36 crab species in GenBank database. *Hapiosquilla harpax* was used as an outgroup for tree rooting (Figure 1). Result showed that *V. yui* was clustered with other Varunidae species (*Helicetient sinensis, Eriocher sinesis,* etc.), suggesting it may also be placed in Grapsoidea. Similarly, it is interesting that these Varunidae species seemed to have close relationship with *Macrophthalmus japonicas*, usually placed in Ocypodidea (Schubart et al. 2006). Therefore, there is still room to reconsideration for the taxonomy of Grapsoidea and Ocypodidea. In conclusion, the complete mitogenome of *V. yui* can provide more essential phylogenetic information of Varunidae.

**Figure 1.**
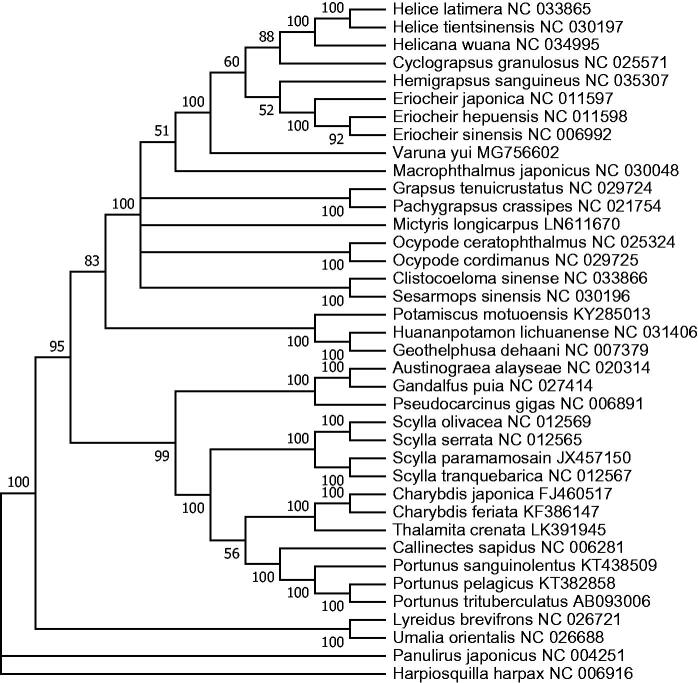
Phylogenetic tree of *V. yui* and related species based on maximum likelihood (ML) method with *Hapiosquilla harpax* as an outgroup.
